# Perceived stress, eating behavior, and overweight and obesity among urban adolescents

**DOI:** 10.1186/s41043-021-00279-2

**Published:** 2021-12-17

**Authors:** S. K. Roy, Khurshid Jahan, Nurul Alam, Rumana Rois, Ambrina Ferdaus, Samina Israt, Md. Rizwanul Karim

**Affiliations:** 1 Bangladesh Breastfeeding Foundation (BBF), Institute of Public Health, Dhaka, Bangladesh; 2 The International Centre for Diarrheal Disease Research (ICDDR, B), Dhaka, Bangladesh; 3 Department of Statistics, Jahangirnagar University, Dhaka, Bangladesh; 4 Department of Community Medicine, Rajshahi Medical College, Rajshahi, Bangladesh

**Keywords:** Adolescent, Mental health, Stressful environment, Overweight, Obesity, DEBQ, PAQ, ASQ

## Abstract

**Background:**

WHO estimated 20% of adolescents (10–19 years) have mental health problems. We examined the prevalence and associated risk predictors of overweight/obesity and perceived stress using eating behaviors and physical activity among school-and-college-going urban adolescents in Bangladesh.

**Methods:**

A cross-sectional study with a multistage sampling technique was employed to select 4609 adolescent students, aged 13–19 years, from all eight Bangladesh divisions during January–June 2019. Data were collected using a self-administered questionnaire containing Turconi Physical Activity Questionnaire (PAQ), Adolescent Stress Questionnaire (ASQ), Dutch Eating Behavior Questionnaire (DEBQ), and Anthropometric measurements. Logistic regression and different association measures assessed relationships among adolescent characteristics.

**Results:**

The major 61.5% of adolescents were in moderate-to-extremely-severe levels of stress, 28.2% were overweight/obese, only 2.7% had a very active lifestyle, and 30.5% had a sedentary lifestyle. Perceived stress was positively and significantly correlated with eating behaviors and body mass index, whereas physical activity was significantly associated with the prevalence of overweight/obesity and high stress. The prevalence of overweight/obesity (53.8%) and high stress (52.5%) was higher in males. Adolescents’ obesity was 2.212 times more likely who had a sedentary lifestyle (95% CI 1.377–3.552), 1.13 times more likely for those who had experienced stress due to school/leisure conflict (95% CI 1.051–1.222), and 1.634 times more likely for those who were tempted by restrained eating behavior (95% CI 1.495–1.786).

**Conclusion:**

Stress on secondary school-and-college-going students needs to be recognized, and strategies need to be developed to improve adolescents’ mental health.

## Introduction

Adolescence, a unique life stage of the socialization process, is defined as a period of human growth and development that occurs after childhood and before adulthood and, according to the United Nations (UN), includes those persons between 10 and 19 years of age [1]. Adolescence is a pivotal time in the life course when there is considerable opportunity for change [2]. Life for many adolescents is a painful tug of war filled with mixed messages and conflicting demands from parents, teachers, friends, family, and oneself [3]. Experiencing stress is especially important in this stage of life, due to the many physiological, cognitive, and social changes which adolescents experience. Although different types of stressful events have been identified, the importance of daily stress has recently come to light [4].

In recent decades, research has shown the negative impact of stress in adolescence, associating it with the presence of internalized or externalized symptomatology and with lower levels of life satisfaction [5, 6]. During adolescence, the levels of stress increase significantly [7]. Adolescents are highly stressed about school performance and attendance, future uncertainty, school/leisure conflict, home life, emerging adult responsibility, romantic pressure, etc. But all of them, school-related stresses are regarded as an important source of adolescent stress [8]. [9] suggested that today’s child has become the unwilling, unintended victim of overwhelming stress; the stress borne of rapid, bewildering social change and constantly rising expectations. Mental ill-health and psychosocial problems typically start during adolescence; unless young girls or boys receive appropriate treatment, the results of these psychosocial problems will continue to affect them as adults [10]. As emotional stress has been linked to health issues, therefore, adolescents need to learn to have confidence in themselves.

Poor eating behaviors, inadequate physical activity, and obesity are some of the most pressing public health problems facing youth and adults [11]. Adolescents who are engaged in disordered eating behaviors find their body mass index (BMI) higher [12]. Increased BMI in adolescence is strongly associated with morbidity and mortality in adulthood [13, 14]. Excess body weight is a major risk factor for mortality and morbidity. The Global Burden of Diseases estimated that the prevalence of obesity has increased over the past three decades at a faster pace [15]. Dyslipidemia, non-alcoholic hepatitis, diabetes mellitus type 2, obstructive sleep apnea, hypertension like comorbidities are linked to adolescent overweight [16]. Overweight and obesity is an issue of epidemic proportions worldwide among adolescents [17].

The report of the National Mental Health System in Bangladesh [10] showed that 16.1% of the adult population (aged 18 years or older) of Bangladesh suffer from some form of mental disorder. BMI was significantly correlated with stress [8]. Most of the studies have indicated that eating behaviors are linked to the BMI [18], nature of food, and psychological outcomes such as depression, anxiety, or body esteem [19]. Increasing physical activity and reducing food intake are widely advocated as the treatment of choice for obesity in all age groups [20]. Psychological stress has been hypothesized to be linked with weight gain through a variety of mechanisms, including effects on behavior (e.g., more intakes of convenient foods and less time for physical activity [21] and emotional reactions such as “comfort eating”) [22]. A few studies regarding the prevalence and risk factors of childhood obesity have been conducted in Bangladesh recently, but focusing only on the influence of stress on BMI. Using a large sample of adolescents from across Bangladesh, this study will examine relationships among adolescents’ perceived stress, eating behavior, physical activity, and overweight and obesity.

## Materials and methods

### Study participants

This cross-sectional study was conducted from January to June 2019 in the high schools and colleges in urban areas covering eight divisions of Bangladesh. Adolescents who were not interested in participating and with any known chronic disease, for example, asthma, diabetes, hypertension, and heart disease, etc., were excluded. Data were collected from students of class 9, 10, 11, and 12 grades aged 13–19 years. A multistage sampling technique was employed to recruit respondents for this study. In the first phase, 32 high schools and colleges were selected using simple random sampling from a prepared list of high schools and colleges having 500 + students covering eight divisions of Bangladesh. Hence, from every eight divisions, we selected four high schools and colleges. Then in each of the selected high schools and colleges, two classes were selected conveniently. In the third phase, 75 students were selected randomly from each selected class, and a pretested structured questionnaire was distributed to all of the selected students in that class. The students were enrolled to ensure an equal number of males and females for all divisions.

### Data collection procedure

A well-trained data collection team from the authors’ institute was employed to collect data by using self-administered questionnaires under the direct supervision of the authors. The questionnaire was pretested in a sample of 20 college-going students before implementation in the final study. The questionnaire contained six sections. Section A contains the questions about the participant’s demographic background, and Section B comprises socioeconomic information of participants. Section C includes Turconi Physical Activity Questionnaire (PAQ). Adolescent Stress Questionnaire (ASQ) and Dutch Eating Behavior Questionnaire (DEBQ) are included in Sections D and E, respectively. Section F contains anthropometric measurements such as body weight, height, mid-upper arm circumference (MUAC), and waist circumference to assess BMI and overall nutritional status. The questionnaire was translated into Bangla and then translated back to English by different persons to assess validity. The data collectors interviewed students about food intake using the 24-h recall method, including types and amounts of foods. After completion of the questionnaire, students were approached for anthropometric measurements. After removing incomplete questionnaires from 4800 respondents, a dataset of 4609 respondents was kept for the final analysis.

### Quality control measures

The study’s quality control was conducted through rigorous training of the field staff and extensive monitoring and supervision of the field activities. The authors’ institute conducted the training by orienting the field staff on questionnaire administration and anthropometry. They were provided with the necessary guidance and supportive supervision. On another level, investigators of the study from the central level provided frequent visits to the field to oversee the activities.

### Measurements of adolescent stress

The 56‐item version of the Adolescent Stress Questionnaire (ASQ), developed and validated by [23], was administered to assess the perceived stress of adolescents. The ASQ is a broadly based instrument and has a good validity to assess adolescent stressor experience [22, 24–29]. This 56‐item scale measuring stress in 10 domains (Home Life, School Performance, School Attendance, Romantic Relationships, Peer Pressure, Teacher Interaction, Future Uncertainty, School/Leisure Conflict, Financial Pressure, and Emerging Adult Responsibility) rated on a 5-point Likert scale: 1 (not at all stressful or is irrelevant to me) to 5 (very stressful). A relative score for each stress component scale was calculated by summing the items belonging to that scale and dividing by the number of scale items. Besides, a stress summary score (perceived stress) was obtained by adding the individual scores of all 56 items [27]. The higher ASQ total score represents the higher level of stress. We also categorized the total stress score (280) using tertiles as low stress = 56–130 points, moderate stress = 130–204 points, and high stress = 204–280 points. In this study, Cronbach’s alphas for the 10 stress domain component scales ranged between 0.80 and 0.86, with an overall Cronbach’s *α* of 0.95 (Table 1), suggesting that the items have relatively high internal consistency.

**Table 1:** Gender differences in the prevalence of self-reported stress and its relationships with BMI and smoking habit among urban school and college-going adolescents

ASQ domain	Items	Cronbach’s *α*	Mean ± SD	Female Mean ± SD	Male Mean ± SD	BMI *ρ* (*p* value)	Smoking habit
Home life	12	0.848	2.61 ± 0.80	2.66 ± 0.80	2.56 ± 0.79	.025 (.088)	0.698^e^
School performance	7	0.820	2.73 ± 0.91	2.76 ± 0.90	2.70 ± 0.92	.037^*^ (.013)	0.654^e^
School attendance	3	0.812	2.31 ± 1.14	2.26 ± 1.13	2.37 ± 1.16	.062^*^ (< .001)	0.451^e^
Romantic relationship	5	0.824	2.23 ± 0.98	2.20 ± 0.96	2.26 ± 1.01	.001(.925)	0.572^e^
Peer pressure	7	0.826	2.13 ± 0.86	2.13 ± 0.84	2.13 ± 0.88	.044^*^ (.003)	0.590^e^
Teacher interaction	7	0.865	2.50 ± 0.97	2.53 ± 0.97	2.46 ± 0.98	.026 (.080)	0.662^e^
Future uncertainty	3	0.862	3.04 ± 1.28	3.07 ± 1.28	2.99 ± 1.28	− .026 (.081)	0.506^e^
School/leisure conflict	5	0.856	2.62 ± 1.04	2.60 ± 1.03	2.64 ± 1.06	.047^*^ (.001)	0.651^e^
Financial pressure	4	0.845	2.62 ± 1.14	2.55 ± 1.12	2.69 ± 1.16	.005 (.716)	0.618^e^
Emerging adult responsibility	3	0.798	2.70 ± 1.15	2.68 ± 1.13	2.71 ± 1.16	− .017 (.243)	0.591^e^
Perceived stress^a^	56	0.951	141.68 ± 38.6	142.15 ± 38.1	141.19 ± 39.1	.034^*^ (.021)	0.866^e^

*Statistically significant at the 0.05 level (2-tailed). *ρ* is the Spearman rank correlation of stress and BMI

^a^Total stress score was produced by the sum of the score from each ASQ’s domain] (Darviri et al. 2014)

^e^Eta (*η*) statistic is calculated for nominal by interval scale of variables

### Eating behavior of adolescent measurements

Eating behavior was assessed using the Dutch Eating Behavior Questionnaire (DEBQ) by Van Strein T. in 1986 for assessing eating behaviors [30]. The DEBQ scale has been extensively used and has proven to be a useful and reliable tool [18, 30–32]. This instrument was also successfully used for Bangladeshi adolescents [8]. It consists of 33 items and three scales, with 13 items assigned to emotional eating (overeating in response to emotions), 10 items to externally induced eating (eating in response to food-related stimuli, regardless of the internal states of hunger and satiety), and 10 items for restrained eating (attempts to refrain from eating). They rated on a 5-point Likert scale: 1 (never) to 5 (very often). For example, in the emotional eating subscale score of 1 means that the adolescent did not eat in response to emotions, and a score of 5 indicated overeating due to emotional statuses such as nervousness, happiness, or excitement. In external eating, the maximum score represented eating in response to stimuli such as color, smell, and taste of the food and 1 score means that they did not pay any attention to these stimuli and only eat when they are starving. As for the last subscale, restrained eating, the maximum score showed that the person has more control over eating behavior and tries to refrain from eating. For this study, Cronbach’s *α* values were 0.841, 0.899, and 0.804 for restrained, emotional, and external eating behaviors, respectively, which indicated significant internal reliability of the DEBQ scales. Scores on each of the three scales were obtained by dividing the sum of items scored by the total number of items on that scale.

### Physical activity level of adolescent measurements

Turconi Physical Activity Questionnaire (PAQ) [33] contains six questions that aim to investigate adolescents’ lifestyle and physical activity levels. All answers were structured to quantify the time spent weekly in physical activity, to investigate the activities spent during the free time, and to quantify the hours spent daily on the computer or in watching TV [33]. Each score ranges from 0 to 3, with the maximum score assigned to the healthiest habit. The total score (18) was divided into tertiles, where the lowest one referred to “sedentary physical level,” the medium one referred to “partially moderate physical level,” and the highest one referred to “active physical level” according to the National Lifestyle Guidelines [34]. The PAQ scale was previously used by the International Centre for Diarrheal Disease Research, Bangladesh (icddr,b) in a survey on adolescents of Bangladesh in 2013.

### Anthropometric measurements

Trained data collectors carried out the weight and height measurements of students in the morning with their school uniform and without shoes. Weight measurement was recorded to the nearest 0.1 kg using electronic portable scales, and height was measured to the nearest 0.1 cm. Waist circumference was measured at the level midway between the lowest rib margin and the iliac crest using a measuring tape. Student’s mid-upper arm circumference was measured with a MUAC tape. Body mass index (BMI) was calculated as weight/height^2^ (kg/m^2^), and adolescents were classified as normal weight/underweight, overweight, and obese according to the International Obesity Task Force age-and gender-specific BMI cutoff values [35]. The BMI cutoff points at age 18, in particular, 18.5 for underweight, 30 for obesity as well as 23 for overweight in Asia [36, 37].

### Statistical analyses

This study aimed to determine the prevalence of obesity and assess the association of obesity with perceived stress, eating behaviors, and physical activities among urban adolescents in Bangladesh. Hence, the primary outcome variable was BMI categories, and the secondary outcomes were stress and eating behaviors of adolescents. Sociodemographic characteristics and physical activities were considered as predictors. The stress summary score of the adolescents was obtained by adding the individual scores of all 56 items from the ASQ. We also categorized the ASQ total stress score as low stress, moderate stress, and high stress. Eating behavior scores on each of the broad three scales (emotional eating, externally induced eating, and restrained eating) were obtained by the average score on that scale from the DEBQ. The PAQ scale introduced three levels of adolescents’ physical activities “sedentary,” “moderately active,” and “active.” Therefore, percentage, mean, and standard deviations were calculated wherever applicable. The associations between overweight and obesity with all other related variables were tested using appropriate measures of associations. Finally, the adolescents’ overweight/obesity and high-stress predictions were explored using the binary logistic regression with corresponding odds ratios (ORs) and 95% confidence intervals (95% CI). A *p* value of less than 0.05 indicates the results are statistically significant at a 5% level of significance, and all tests were two-tailed. All statistical analyses were performed using IBM SPSS Statistics for Windows, version 26.0 (IBM Corp., Armonk, NY). Only the performance of logistic regression models was illustrated with the receiver operating characteristic (ROC) and the k-fold cross-validation using the Scikit-learn module in Python version 3.7.3.

## Results

A total of 4800 eligible high school and college-going adolescents in urban areas completed the self-structured questionnaires, of whom 191 participants were excluded for incompleteness. Among 4609 participants, 51.1% (*n* = 2355) were females and 48.9% (*n* = 2254) were males. The majority of adolescents had HSC or below educated fathers 70.4% (*n* = 3247) and mothers 82.3% (*n* = 3793), fathers engaged in business 41.5% (*n* = 1914) and housewife mothers 88.7% (*n* = 4088), were living in a city 62.8% (*n* = 2893). More than one-fourth of participants were overweight and obese 28.2% (*n* = 1302), 48.1% (*n* = 2219) were normal weight, and 23.6% (*n* = 1088) were underweight. The major 55.7% of adolescents were in moderate stress, followed by 38.5% in a low stress, while only 5.7% were in high stress. Therefore, the major 61.5% of adolescents were in moderate-to-extremely-severe levels of stress. Among the highly stressed adolescents, 28.5% (*n* = 79) were overweight and obese, 8.3% (*n* = 22) were cigarette smokers, 38.1% (*n* = 101) had a sedentary lifestyle, and were more fascinated (2.84 ± 0.882) by external eating behavior (Table 3). Overall, urban adolescents had a high tendency for external eating (2.48 ± 0.747) behavior, 66.8% involved in a moderately active lifestyle (Table 2).

**Table 2:** Distribution of variables and relationship with eating behaviors and physical activities level scores among urban school and college-going adolescents

Characteristics	Total 4609 *n* (%)	Eating behaviors			Physical activity and lifestyle			
		Restrained eating Mean ± SD/*ρ* (*p* value)	Emotional eating Mean ± SD/*ρ* (*p* value)	External eating Mean ± SD/*ρ* (*p* value)	Sedentary *n* (%)	Moderately active *n* (%)	Active *n* (%)	*χ*^2^ (*p* value)
Mean ± SD	–	2.07 ± .737	1.76 ± .713	2.48 ± .747	–	–	–	
*Gender*		.090 ^e^	.083 ^e^	.053 ^e^				
Female	2355 (51.1)	2.14 ± .753	1.70 ± .719	2.44 ± .728	779 (33.1)	1521 (64.6)	55 (2.3)	16.657^*^ (< .001)
Male	2254 (48.9)	2.01 ± .715	1.82 ± .701	2.52 ± .766	627 (27.8)	1556 (69.0)	71 (3.1)	
*Age Categories*		.072 ^e^	.091 ^e^	.058 ^e^				
< 16	506 (11.0)	2.22 ± .736	1.90 ± .821	2.60 ± .727	122 (24.1)	374 (73.9)	10 (2.0)	38.819^*^ (< .001)
16–17	2503 (54.3)	2.05 ± .734	1.71 ± .665	2.47 ± .738	789 (31.5)	1668 (66.6)	46 (1.8)	
18 +	1600 (34.7)	2.07 ± .738	1.80 ± .739	2.45 ± .765	495 (30.9)	1035 (64.7)	70 (4.4)	
*Residence type*		.073 ^e^	.040 ^e^	.015 ^e^				
City	2893 (62.8)	2.12 ± .758	1.74 ± .708	2.49 ± .746	890 (30.8)	1924 (66.5)	79 (2.7)	0.246 (.884)
Town	1716 (37.2)	2.01 ± .697	1.80 ± .720	2.47 ± .751	516 (30.1)	1153 (67.2)	47 (2.7)	
*Father’s education*		.071 ^e^	.100 ^e^	.088 ^e^				
HSC and below	3247 (70.4)	2.04 ± .707	1.71 ± .673	2.44 ± .742	979 (30.2)	2194 (67.6)	74 (2.3)	9.843^*^ (.007)
Graduate and above	1362 (29.6)	2.16 ± .799	1.87 ± .789	2.58 ± .751	427 (31.4)	883 (64.8)	52 (3.8)	
*Mother’s education*		.057 ^e^	.043 ^e^	.047 ^e^				
HSC and below	3793 (82.3)	2.06 ± .718	1.74 ± .699	2.46 ± .743	1154 (30.4)	2552 (67.3)	87 (2.3)	16.095^*^ (.000)
Graduate and above	816 (17.7)	2.17 ± .817	1.83 ± .767	2.56 ± .763	252 (30.9)	525 (64.3)	39 (4.8)	
*Father’s occupation*		.058 ^e^	.099 ^e^	.050 ^e^				
Service	1543 (33.5)	2.11 ± .769	1.83 ± .764	2.53 ± .762	498 (32.3)	988 (64.0)	57 (3.7)	12.854^*^ (.012)
Business	1914 (41.5)	2.09 ± .733	1.77 ± .727	2.46 ± .735	565 (29.5)	1305 (68.2)	44 (2.3)	
Retired or others	1152 (25.0)	2.00 ± .696	1.65 ± .593	2.44 ± .746	343 (29.8)	784 (68.1)	25 (2.2)	
*Mother’s occupation*		.037 ^e^	.074 ^e^	.079 ^e^				
Service	426 (9.2)	2.16 ± .789	1.88 ± .811	2.59 ± .804	124 (29.1)	275 (64.6)	27 (6.3)	28.356^*^ (< .001)
Housewife	4088 (88.7)	2.07 ± .730	1.74 ± .692	2.46 ± .735	1247 (30.5)	2747 (67.2)	94 (2.3)	
Business or others	95 (2.1)	2.03 ± .779	1.99 ± 1.00	2.79 ± .903	35 (36.8)	55 (57.9)	5 (5.3)	
Perceived stress^a^		.106^*^(.000)	.108^*^(.000)	.295^*^(.000)	–	–	–	.043^e^
*Stress categories*		.102 ^e^	.128 ^e^	.248 ^e^				
Low stress	1776 (38.5)	1.98 ± .703	1.64 ± .619	2.26 ± .718	524 (29.5)	1197 (67.4)	55 (3.1)	10.583^*^ (.032)
Moderate stress	2568 (55.7)	2.12 ± .738	1.83 ± .751	2.60 ± .710	781 (30.4)	1719 (66.9)	68 (2.6)	
High stress	265 (5.7)	2.21 ± .883	1.79 ± .804	2.84 ± .882	101 (38.1)	161 (60.8)	3 (1.1)	
BMI		.201^*^(.000)	.061^*^ (.000)	.055^*^ (.000)	–	–	–	
*BMI categories*		.209 ^e^	.075 ^e^	.051 ^e^				
Underweight	1088 (23.6)	1.84 ± .620	1.67 ± .630	2.43 ± .718	327 (30.1)	736 (67.6)	25 (2.3)	9.871^*^ (.043)
Normal weight	2219 (48.1)	2.07 ± .747	1.76 ± .703	2.48 ± .759	667 (30.1)	1475 (66.5)	77 (3.5)	
Overweight/obesity	1302 (28.2)	2.27 ± .754	1.82 ± .783	2.53 ± .749	412 (31.6)	866 (66.5)	24 (1.8)	
*Physical activity and lifestyle*	.040 ^e^	.066 ^e^	.022 ^e^					
Sedentary	1406 (30.5)	2.04 ± .752	1.73 ± .705	2.50 ± .748	–	–	–	
Moderately active	3077 (66.8)	2.09 ± .744	1.76 ± .709	2.47 ± .742	–	–	–	
Active	126 (2.7)	2.19 ± .895	2.02 ± .811	2.47 ± .863	–	–	–	
*Smoking habit*		.047 ^e^	.022 ^e^	.017 ^e^				
Yes	207 (4.5)	1.91 ± .674	1.83 ± .739	2.54 ± .806	68 (32.9)	134 (64.7)	5 (2.4)	0.604 (.739)
No	4402 (95.5)	2.08 ± .739	1.75 ± .711	2.48 ± .745	1338 (30.4)	2943 (66.9)	121 (2.7)	

^*^Statistically significant at the 0.05 level (2-tailed). *ρ* is the Spearman rank correlation of stress and BMI. ^a^Total stress score was produced by the sum of the score from each ASQ’s domain (Darviri et al. 2014).^**e**^ Eta (*η*) statistic is calculated for nominal by interval scale of variables

Table 1 reveals summary information of the 56-item ASQ scores, and perceived stress, and their relationship with the outcome variable BMI, the smoking habit of adolescents, and two essential demographic variables—sex and age. Adolescents reported the highest levels of stress from future uncertainty and school performance, whereas the lowest levels of stress were seen from peer pressure and romantic relationships. Similar highest and lowest levels of stress domains were reflected for girls and boys adolescents. The average perceived stress of urban adolescents was 141.68 points, which advocates a moderate stress category. Considering the average perceived stress, we found that female adolescents (142.15) are a bit more stressed than males (141.19). To assess the relationship between adolescents’ stress with their BMI, age, and family income, Spearman’s rank correlation coefficients (*ρ*) with *p* values are also reported in Table 1. School performance, school attendance, peer pressure, and school/leisure conflict were the four major stress domains of adolescents that had a significant positive correlation with their BMI. Perceived stress of adolescents was positively significantly correlated with their BMI. Observing the evaluated value of the Eta statistic, which is a more appropriate measure of association between a categorical variable and an interval scale variable that ranges from 0 to 1, with 0 indicating no association and values close to 1 indicating a high degree of association [38], a very strong association was also revealed for the smoking habits of adolescents with their all stress domains and perceived stress.

Eating behaviors were assessed by using the summated scores of three individual subscales, where higher scores indicate higher levels of restrained, external, and emotional eating behaviors. Urban adolescents had a high tendency for external eating (2.48 ± 0.747) behavior, compared to restrained (2.07 ± 0.737) and emotional eating (1.76 ± 0.713) behaviors, which manifested in all categories that are illustrated in Table 2.

All the eating behaviors were significantly positively correlated with adolescents’ perceived stress and BMI, which emphasizes adolescents who are stressed are more likely to have restrained, emotional, and external eating behaviors that increase their BMI. Adolescents who are physically more active and had a healthy lifestyle had less tendency for external eating behavior (*ρ* = − 0.007, *p* = 0.632). Examining the values of Eta, a significant association was revealed for emotional and external eating behaviors of adolescents with their stress categories. Though common thinking about emotional eating is that it has often been linked to overweight and/or obesity, we found only restrained eating behavior had a significant association (*η* = 0.169) for adolescents with overweight and obesity.

Table 2 also exhibits the frequency distribution of physical activity level according to sociodemographic characteristics of urban adolescents. Only 2.7% of the respondents had a very active lifestyle, while about 30.5% showed a sedentary physical level and the remaining large percentage of respondents 66.8% were moderately active. Though most adolescents had moderate physical activity levels, more than two-thirds of the students (80.7%) preferred spending many hours in sedentary activities (watching television, using the computer, listening to music, reading a book), which is a typical adolescent’s habit. Significant associations were observed in adolescents’ gender (*χ*^2^ = 16.657, *p* < 0.05) and age categories (*χ*^2^ = 38.819, *p* < 0.05), fathers’ education (*χ*^2^ = 9.843, *p* < 0.05) and occupation (*χ*^2^ = 12.854, *p* < 0.05), mothers’ education (*χ*^2^ = 16.095, *p* < 0.05) and occupation (*χ*^2^ = 28.356, *p* < 0.05), BMI categories (*χ*^2^ = 9.871, *p* < 0.05), and stress categories (*χ*^2^ = 10.583, *p* < 0.05) with their physical activities and lifestyle. Though a significant association was expected between adolescents’ physical activities with their perceived stress, we did not observe any significant association with that in our findings. Smoking habit was also not significantly associated with adolescents’ physical activities. Moreover, adolescents’ physical activity was significantly associated with a higher prevalence of obesity (*χ*^2^ = 6.050, *p* < 0.05).

The prevalence of overweight/obesity of urban adolescents was estimated by categorizing BMI into underweight (BMI < 18.5), normal weight (18.5 ≤ BMI ≤ 23), and overweight and obesity (BMI > 23), and the result is summarized in Table 3. The prevalence rates of underweight, normal weight, and overweight/obesity among urban adolescents were 23.6%, 48.2%, and 28.2%, respectively. A higher proportion (53.8%) of male adolescents were overweight and obese than females (Table 3). Among the urban adolescents, gender, age, father’s education and occupation, and physical activity and lifestyle were significantly associated with the prevalence of overweight/obesity (Table 3). As stress is also the outcome variable for this study, therefore, to calculate the prevalence of high stress, we used stress categories (low = 56–130, moderate = 130–204, and high = 204–280). Using the stress categories, we observed that the major 55.7% of adolescents were in moderate stress, followed by 38.5% in a low stress, while only 5.7% in high stress. Therefore, the major 61.5% of adolescents were in moderate-to-extremely-severe levels of stress, indicating an alarming situation. Adolescents’ smoking habits, all the eating behaviors (restrained, external, and emotional), and physical activity and lifestyle were significantly associated with the prevalence of high stress (Table 3).

**Table 3:** The relationships of perceived stress, eating behaviors, and physical activity with overweight/obesity and high stress, controlling for sociodemographic characteristics

Variables	Overweight/obesity (*n* = 1302; 28.2%)			High Stress (*n* = 265; 5.7%)		
	Yes (%)	*χ*^2^ (*p *val.)	OR (*p* value) (95% CI)	Yes (%)	*χ*^2^ (*p *val.)	OR (*p* value) (95% CI)
*Gender*						
Female	602 (46.2)	17.146^*^ (.000)	0.713^*^ (< .001) (0.623–0.816)	126 (47.5)	1.417 (.234)	0.908 (.469) (0.700–1.178)
Male	700 (53.8)		Ref	139 (52.5)		ref
*Age categories*						
< 16	144 (11.1)	9.545^*^ (.008)	0.784^*^ (.039) (0.623–0.988)	25 (9.4)	1.085 (.581)	0.819 (.404) (0.512–1.309)
16–17	663 (50.9)		0.799^*^ (.002) (0.692–0.922)	151 (57.0)		1.088 (.546) (0.827–1.433)
18 +	495 (38.0)		Ref	89 (33.6)		ref
*Residence type*						
City	843 (64.7)	3.038 (0.081)	1.082 (.278) (0.939–1.246)	168 (63.4)	0.047 (0.828)	1.012 (.929) (0.775–1.321)
Town	459 (35.3)		Ref	97 (36.6)		ref
*Father’s education*						
HSC and below	845 (64.9)	26.839^*^ (.000)	0.702^*^ (< .001) (0.591–0.835)	181 (68.3)	0.623 (.430)	–
Graduate and above	457 (35.1)		Ref	84 (31.7)		–
*Father’s occupation*						
Service	475 (36.5)	11.218^*^ (.004)	1.158 (.116) (0.964–1.392)	82 (30.9)	1.174 (.556)	–
Business	540 (41.5)		1.134 (.151) (0.955–1.346)	118 (44.5)		–
Retired and others	287 (22.0)		Ref	65 (24.5)		–
*Mother’s education*						
HSC and below	1048 (80.5)	4.053^*^ (.044)	1.137 (.212) (0.929– 1.391)	219 (82.6)	0.023 (.879)	–
Graduate and above	254 (19.5)		Ref	46 (17.4)		–
*Smoking habit*						
Yes	51 (3.9)	1.395 (.238)	–	22 (8.3)	9.518^*^ (.002)	1.916^*^ (.008) (1.187–3.092)
No	1251 (96.1)		–	243 (91.7)		ref
*Physical activity and lifestyle*						
Sedentary	412 (31.6)	6.050^*^ (.049)	2.212^*^ (.001) (1.377–3.552)	101 (38.1)	9.492^*^ (.009)	3.394^*^ (.042) (1.047–11.002)
Moderately active	866 (66.5)		2.023^*^ (.003) (1.270– 3.224)	161 (60.8)		2.473 (.129) (0.768–7.961)
Active	24 (1.8)		Ref	3 (1.1)		ref
*Stress categories*						
Low stress	485 (37.3)	1.397 (.497)	–	–	–	–
Moderate stress	738 (56.7)		–	–		–
High stress	79 (6.1)		–	–		–
*BMI categories*						
Underweight	–	–	–	61 (23.0)	0.340 (.844)	1.102 (.595) (0.770–1.578)
Normal weight	–		–	125 (47.2)		1.021 (.891) (0.759–1.374)
Overweight/obesity	–		–	79 (29.8)		ref
*Stress domains*						
Future uncertainty $\overline{X} \pm {\text{SD}}$	2.96 ± 1.287	0.039^e^	.926^*^ (.013) (0.872–0.984)	–	–	–
School/leisure conflict $\overline{X} \pm {\text{SD}}$	2.69 ± 1.065	0.042^e^	1.133^*^ (.001) (1.051–1.222)	–	–	–
Emerging adult resp. $\overline{X} \pm {\text{SD}}$	2.64 ± 1.129	0.028^e^	0.921^*^ (.018) (0.861–0.986)	–	–	–
Restrained eating $\overline{X} \pm {\text{SD}}$	2.07 ± 0.747	0.169^e^	1.634^*^ (< .001) (1.495–1.786)	2.21 ± 0.883	0.044 ^e^	1.288^*^ (.003) (1.090–1.522)
Emotional eating $\overline{X} \pm {\text{SD}}$	1.82 ± 0.704	0.055^e^	–	1.79 ± 0.804	0.011^e^	–
External eating $\overline{X} \pm {\text{SD}}$	2.53 ± 0.749	0.043^e^	–	2.84 ± 0.882	0.118 ^e^	1.883^*^ (< .001) (1.601–2.214)

^*^ Statistically significant at the 0.05 level (2-tailed). ^**e**^ Eta (*η*) statistic is calculated for nominal by interval scale of variables

Overweight/obese participants were more likely to be male (53.8%), be aged 16–17 years (50.9%), had less educated (HSC and below) fathers (64.9%) and mothers (80.5%), had businessman fathers (41.5%), involve in a moderately active physical activity level (66.5%), and be in moderate-to-extremely-severe levels of stress (62.7%). Furthermore, highly stressed participants were also more likely to be male (52.5%), be aged 16–17 years (57%), live in a city (63.8%), had less educated (HSC and below) fathers (68.3%) and mothers (82.6%), had businessman fathers (44.5%) and housewife mothers (91.3%), be a non-cigarette smoker (91.7%), engage in a moderately active lifestyle (60.8%), and be normal weight (47.5%). Investigating the calculated values of the Eta statistic, only a significant association was observed for restrained eating behavior of adolescents with the prevalence of overweight/obesity. In contrast, external and emotional eating behaviors were significantly associated with the prevalence of high stress. All the significant associated variables with some control variables were only considered in the logistic regression analysis to predict the prevalence of overweight/obesity and high stress of adolescents. Considering the sociodemographic characteristics (gender, age, residence) as control variables, and father’s education, father’s occupation, mother’s education, physical activity and lifestyle, three stress scales (future uncertainty, school/leisure conflict, and emerging adult responsibility), and restrained eating behavior as predictors in the logistic regression model to predict the prevalence of overweight/obesity. Conversely, the sociodemographic characteristics (gender, age, residence, BMI categories) as control variables, along with the significantly associated variables, for instance, smoking habit, physical activity and lifestyle, and external and restrained eating behaviors were used as predictors in the logistic regression model to predict the prevalence of high stress.

Logistic regression analysis further revealed that female adolescents were less likely (OR 0.713, 95% CI 0.636–0.945, *p* < 0.05) to be obese. Adolescents aged less than 16 years (OR 0.784, 95% CI 0.623–0.988, *p* < 0.05) and aged 16–17 years (OR 0.799, 95% CI 0.692–0.922, *p* < 0.05) were less likely to be overweight and obese than those aged 18 years or older. Respondents who had less educated fathers less likely to be overweight and obese than those who had higher educated fathers (OR 0.702, 95% CI 0.591–0.835, *p* < 0.05). Adolescents who had service holder fathers (OR 1.158, 95% CI 0.964–1.392, *p* = 0.116) and businessman fathers (OR 1.134, 95% CI 0.955–1.346, *p* = 0.151) were more likely to be overweight and obese than those who had retired fathers. Students who had a sedentary lifestyle were 2.212 times more likely (OR 2.212, 95% CI 1.377–3.552, *p* < 0.05) and who had a moderate active lifestyle were 2.023 times more likely (OR 2.023, 95% CI 1.270–3.224, *p* < 0.05) to be overweight and obese than those who had a very active lifestyle. Respondents who had experienced stress due to future uncertainty, school/leisure conflict, and emerging adult responsibility were statistically significant as the predictors of overweight and obese. Respondents who had experienced stress due to school/leisure conflict had 1.133 times higher risk of gaining weight and becoming overweight and obese (OR 1.133, 95% CI 1.051–1.222, *p* < 0.05). Restrained eating behavior was a significant predictor for overweight and obesity (OR 1.634, 95% CI 1.495–1.786, *p* < 0.05) of urban adolescents.

To predict the prevalence of high stress, logistic regression model also illustrated that female adolescents were less likely (OR 0.908, 95% CI 0.700–1.178, *p* = 0.469) to be high stressed. Cigarette smoker adolescents were 1.916 times more likely (OR 1.916, 95% CI 1.187–3.092, *p* < 0.05) to be high stressed than non-smokers. Adolescents who had a sedentary lifestyle were 3.394 times more likely (OR 3.394, 95% CI 1.047–11.002, *p* < 0.05), and who had a moderate active lifestyle were 2.473 times more likely (OR 2.473, 95% CI 0.768–7.961, *p* = 0.129) to be high stress than those who had a very active lifestyle. Respondents who were more fascinated by the restrained eating behavior were 1.288 times more likely to be highly stressed (OR 1.288, 95% CI 1.090–1.522, *p* < 0.05) and 1.883 times more likely to be highly stressed (OR 1.883, 95% CI 1.601–2.214, *p* < 0.05) who had more tendency for external eating behavior.

In predicting the prevalence of overweight/obesity, gender, age categories, father’s education, physical activity and lifestyle, stress scales (future uncertainty, school/leisure conflict and emerging adult responsibility), and restrained eating behavior of adolescents were found significant in the logistic regression model. In contrast, smoking habit, physical activity and lifestyle, restrained and external eating behaviors were observed significantly to estimate the prevalence of high stress of adolescents. Table 3 expresses the unadjusted logistic models (with unadjusted or crude odds ratio [39, 40]) for predicting the prevalence of overweight/obesity as well as high stress of urban adolescents.

**Fig. 1:**
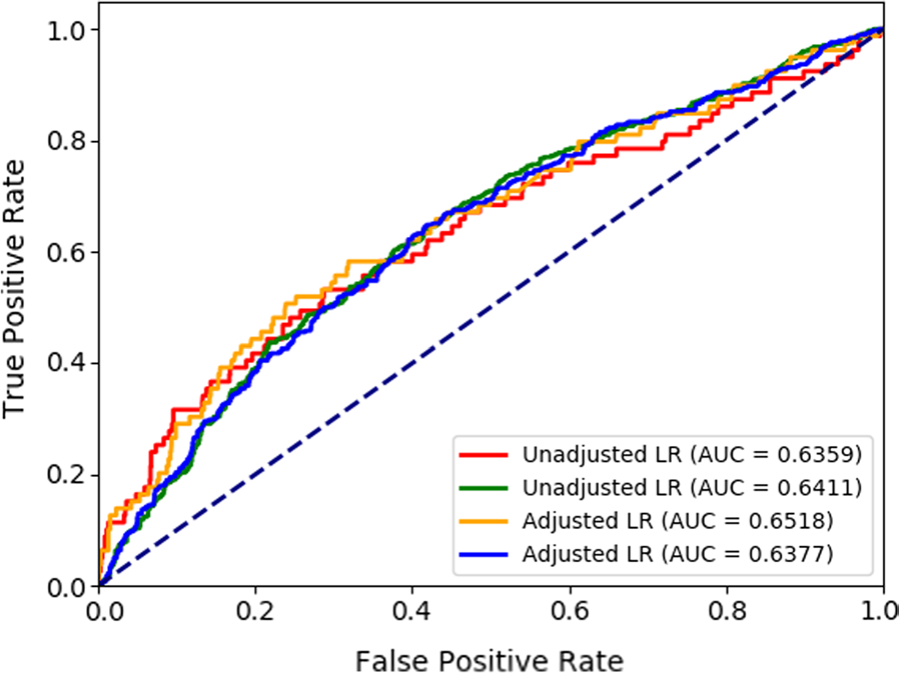
The ROC curves to predict high stress using the unadjusted logistic regression model in red and the adjusted model in orange and to predict overweight/obesity the unadjusted model in green and the adjusted model in blue

Adjusted logistic models (with adjusted odds ratio [39, 40]) were also run using the Scikit-learn module in Python 3.7.3, considering 70% observations as training data and 30% observation as test data with the random seed 4371. The area under the ROC curve (AUC) was estimated and is plotted in Fig. 1. To predict the prevalence of high stress using an unadjusted model—the AUC was 0.6359 with an accuracy score of 0.9429, and for the adjusted model—the slightly improved AUC was 0.6518 with the same accuracy score of 0.9429. Moreover, to estimate overweight and obesity prevalence using unadjusted and adjusted logistic regression models, the AUC was 0.6411 with an accuracy score of 0.7267, and the AUC was 0.6377 with an accuracy score of 0.7238, respectively. To evaluate the performance of unadjusted and adjusted logistic regression models based on several runs, we used the k-fold cross-validation for 10-fold, 20-fold, and 30-fold repetitions using the Scikit-learn module in Python 3.7.3 with random seed 1 and shuffle argument ‘True’, and the results are arranged in Table 4. The unadjusted and adjusted logistic regression models had performed almost equally with the similar accuracy scores, and the similar uncertainty (standard error) of the parameter estimates to predict stress and overweight/obesity (Table 4).

**Table 4:** Result of K-fold cross-validation of the unadjusted and adjusted logistic regression models to predict stress and overweight/obesity

Models	10-fold		20-fold		30-fold	
	MAcc	SE	MAcc	SE	MAcc	SE
Unadjusted LR to predict stress	0.9425	0.0081	0.9425	0.0115	0.9425	0.0162
Adjusted LR to predict stress	0.9425	0.0081	0.9425	0.0115	0.9425	0.0162
Unadjusted LR to predict obesity	0.7110	0.0227	0.7112	0.0383	0.7119	0.0445
Adjusted LR to predict obesity	0.7129	0.0237	0.7129	0.0368	0.7132	0.0431

*MAcc* mean of accuracy scores from each fold, *SE* standard error of accuracy scores

Evaluating the performance of adjusted and unadjusted logistic models using the accuracy scores, the ROC (Fig. 1), and the *k*-fold cross-validation (Table 4), the gain in adjusted models’ precisions is minimal. Therefore, the result of unadjusted models in Table 3 remains tenable.

## Discussion

Stress is not a psychiatric diagnosis, but it is closely linked to mental health conditions including depression, anxiety, psychosis, and post-traumatic stress disorder (PTSD) [41]. Mental health conditions account for 16% of the global burden of disease and injury of adolescents. The consequences of not addressing adolescent mental health conditions extend to adulthood, impairing both physical and mental health and limiting opportunities to lead fulfilling lives as adults [42]. Despite widespread and increasing awareness of the mental health challenges facing adolescents, their needs in this area are largely unmet and particularly in developing countries [2]. Using the meta-analysis, a review study [43] demonstrates the increasing prevalence of overweight and obesity among children and adolescents in both urban and rural areas of Bangladesh, but adolescents’ stress was silenced. Hence, this study was conducted to investigate the association between stress, eating behavior, and physical activity with BMI among urban adolescent students. It was conducted in randomly selected schools and colleges in eight Divisions of Bangladesh. For ease of our discussion, we have referred BMI categories into three groups: underweight, normal weight and overweight and obesity, and stress categories also into three groups: low, moderate, and high stress.

The study findings reveal that more than half of adolescents were in moderate-to-extremely-severe levels of stress, two-seventh were overweight and obese, one-fourth were underweight, only one-fifty had a very active lifestyle, while more than one-fourth had a sedentary lifestyle. Among the highly stressed adolescents, almost two-seventh were overweight and obese, less than one-tenth were cigarette smokers, more than two-sixth had a sedentary lifestyle, and were more fascinated by external eating behavior. Perceived stress was positively and significantly correlated with all three scales of adolescents’ eating behaviors and BMI, whereas physical activity was significantly associated with a higher prevalence of overweight/obesity and high stress. Adolescents reported the highest stress levels from future uncertainty and school performance, whereas the lowest stress levels were seen from peer pressure and romantic relationships. Similar scenarios were reflected for both boys’ and girls’ adolescents.

Urban adolescents had a high tendency by external eating behavior, compared to restrained and emotional eating behaviors, which manifested in all categories. Eating behaviors were significantly positively correlated with adolescents’ perceived stress and BMI, which emphasizes adolescents who are stressed are more likely to have restrained, emotional, and external eating behaviors that increase their BMI. Examining the values of Eta, a significant association was revealed for emotional and external eating behaviors of adolescents with their stress categories. Though common thinking about emotional eating is that it has often been linked to overweight and obesity, we found only restrained eating behavior had a significant association for adolescents with overweight and obesity.

Few of the respondents had a very active lifestyle, while more than one-fourth showed a sedentary physical activity level, and the remaining large percentage (more than three-fifth) of respondents were moderately active, not consistent with a healthy lifestyle. Adolescents’ physical activity and lifestyle were significantly associated with their gender, age categories, parents’ education and occupation, stress and BMI categories. Though expected, no significant association existed between adolescents’ lifestyles with their perceived stress. Moreover, adolescents’ physical activity was significantly associated with a higher prevalence of obesity.

Among the urban adolescents, gender, age, father’s education and occupation, mother’s education, physical activity and lifestyle, and restrained eating behavior were significantly associated with the prevalence of overweight and obesity. However, adolescents’ gender, age, father’s education, physical activity and lifestyle, stress due to future uncertainty, school/leisure conflict and emerging adult responsibility, and restrained eating behavior were the significant predictors for estimating the prevalence of overweight and obesity. The prevalence of overweight/obesity was higher in males than in females. Adolescents’ obesity was five-seventh times less likely among females than males, seven-tenths times less likely among respondents with less educated fathers, above two times more likely for those who had a sedentary lifestyle, and two times more likely for those who had a moderately active lifestyle than those who had a very active lifestyle, more likely for those who had experienced stress due to school/leisure conflict, but less likely for those who had experienced stress due to future uncertainty and emerging adult responsibility, and more likely among respondents with restrained eating behavior.

The prevalence of high stress among urban adolescents was significantly associated with their smoking habit, physical activity and lifestyle, and external eating behavior, whereas significant predictors for estimating the prevalence of high stress were smoking habit, physical activity and lifestyle, restrained and external eating behaviors. Adolescents’ high stress was about two times more likely who smoke cigarettes, about three and half times more likely who had a sedentary lifestyle, and about two times more likely for those who were tempted by external eating.

Although research on adolescents’ perceived stress and overweight/obesity using ASQ, DEBQ, and PAQ has attracted researchers’ attention globally, it is very much understudied in Bangladesh. Consequently, there is a knowledge gap in Bangladesh which is why the present study assessed adolescents’ perceived stress and overweight/obesity using their physical activity and lifestyle, eating behaviors, and ten different stress scales among secondary school and college-going urban students more generally across the country. Compared to the prevalence of overweight/obesity and perceived stress using ASQ, DEBQ, and PAQ globally, a cross-sectional study was investigated with adolescents from six European cities involved in the Healthy Lifestyle in Europe by Nutrition in Adolescence. The result showed that while girls reported systematically higher levels of stress compared with boys, their stress profiles were similar, with the highest levels for school-related stress followed by future uncertainty. Only in girls, perceived stress was significantly associated with increased measures of general and abdominal adiposity. In boys, no relationship between perceived stress and adiposity measures was observed [27]. A study was conducted among Japanese university students to investigate the association of anthropometric status, perceived stress, and personality traits with eating behavior. The result shows that associations between eating behavior and anthropometric status or psychological factors are different by each eating behavior, which is partly influenced by gender differences [44].

In a longitudinal study of adolescents in London, prospective associations between perceived stress and changes in waist circumference and BMI were examined. Perceived stress in any year was not related prospectively to increases in waist circumference or BMI. However, waist circumference and BMI were significantly higher in the moderate and higher stress groups than the lower stress group across the whole 5-year period [45]. A study in Los Angeles showed no differences in emotional eating between normal-weight and overweight students. Perceived stress was indeed a significant correlate of emotional eating, but emotional eating is not an issue only for overweight and obese persons. This study shows that some children in this population at increased risk for obesity and related chronic disease have already incorporated emotional eating as a learned response to stress by the time they enter into adolescence [46].

Common mental health problems and overweight and obesity of adolescents have emerged as the major public health concerns and can contribute to adult physical and mental health risks. Moreover, this risk could also increase the burden of chronic non-communicable diseases (NCDs) of adults, such as diabetes, hypertension, and cardiovascular diseases [43]. This study reveals that among the respondents, more than half of adolescents were in moderate to extremely severe levels of stress, two-seventh were overweight and obese, only one-fifty had a very active lifestyle and were more fascinated by external eating behavior. This situation is very alarming, as adolescence is a period of physical and mental development. High stress and obesity can cause a harmful effect on the mental and physical development of adolescents. Many awareness programs are being conducted by the government and non-government organizations, which need to identify their impact.

This study was conducted among urban adolescents who are studying in school and colleges, but those adolescents are missing who are not going to school or colleges and those who are in rural households. This limitation of our study could be abolished further by considering rural and urban adolescents to explore the complete and accurate picture of adolescents’ physical and mental status. Another limitation of this study is our findings was unable to illustrate any aspect regarding the COVID-19 pandemic on the perceived stress, eating behavior, and overweight/obesity among urban adolescents. Though the pandemic has a significant influence on our study topic, conducting research during the pre-pandemic context is responsible for this limitation. A reasonable number of researchers is carried out to address that issue during the COVID-19 pandemic [47–49].

The health system of Bangladesh is still primarily focused on maternal and child health and communicable diseases; there is a serious paucity of credible policy and programmatic development for adolescents’ perceived stress, overweight and obesity, and nutritional status. The more we understand mental and physical status among adolescents, the better we can tackle adolescents’ performance, thereby improving adults’ mental and physical health. Counseling and support from parents, teachers, and friends can be very effective in overcoming this situation.

## Conclusion

The secondary and higher secondary education period has been regarded as a stressful environment for students. Adolescents reported the highest stress level from future uncertainty and school performance, whereas a low level of stress from peer pressure and romantic relationship. Body mass index was significantly associated with all three eating behaviors and perceived stress of urban adolescents, whereas physical activity was significantly associated with BMI and stress categories as well as the prevalence of overweight/obesity and high stress. Besides stress and eating behavior, physical activity also plays a vital role in determining the body mass index. This study found that more than half of adolescent boys or girls were in moderate-to-extremely-severe levels of stress. Compared with 20% of adolescents estimated by WHO having mental health problems, the situation found in our study is very alarming, indicating the need to undertake necessary steps.

### Recommendation

Stress on secondary school and college students needs to be recognized, and strategies need to be developed to improve the mental and social health of adolescents. Student counseling on stress factors, increase opportunities for play and recreation, increased support from teachers and parents could be effective. Intervention studies can be undertaken to see the effect of counseling and support from parents, teachers, and friends. Further research needs to be conducted on adolescents who are not going to educational institutions and who are residing in the rural area.

## Data Availability

The datasets that support the findings of this study are available on request.
